# The Prevalence of Anemia and Diagnostic Usefulness of Ferritin and Hepcidin in Antiphospholipid Syndrome and Systemic Lupus Erythematosus Patients

**DOI:** 10.3390/diseases14030101

**Published:** 2026-03-11

**Authors:** Natasa Stanisavljevic, Ljudmila Stojanovich, Aleksandra Djokovic, Violeta Dopsaj, Neda Milinkovic, Dusica Mrdaković, Olivera Markovic, Marija Zdravkovic, Dragomir Marisavljevic

**Affiliations:** 1 University Clinical Center “Bezanijska Kosa”, 11080 Belgrade, Serbiaom1801968@gmail.com (O.M.); sekcija.kardioloska@gmail.com (M.Z.); 2 Medical Faculty, University of Belgrade, 11000 Belgrade, Serbia; 3 Special Hospital “Zutic”, 11000 Belgrade, Serbia; ljudmila_stojanovich@yahoo.com; 4 Faculty of Pharmacy, University of Belgrade, 11000 Belgrade, Serbianeda.milinkovic@pharmacy.bg.ac.rs (N.M.); dusicamrdakovic87@gmail.com (D.M.); dragomir.marisavljevic@gmail.com (D.M.)

**Keywords:** antiphospholipid syndrome, systemic lupus erythematosus, anemia, hepcidin, ferritin

## Abstract

Background: Anemia is common among patients with antiphospholipid syndrome (APS). It can persist alone (primary APS—pAPS) or with another associated disease (secondary APS—sAPS), predominantly systemic lupus erythematosus (SLE). There are no systematic reviews addressing the type of anemia (iron deficiency without anemia—IDWA, iron deficiency—IDA, and anemia of chronic disease—ACD) in these patients. Objectives: This study aimed to assess the type of anemia and to compare the usefulness of common diagnostic anemia parameters and their mutual relations. Methods: A cross-sectional study involving 163 patients was conducted at the University Clinical Center Bezanijska kosa from June 2022 to June 2024, including 79 patients with pAPS, 47 with sAPS and 37 patients diagnosed with SLE. We compared the usefulness of iron metabolism markers (serum iron—Fe; total iron-binding capacity—TIBC; ferritin; hepcidin) in the presence of inflammatory markers such as high-sensitivity (hsCRP) and IL6 in determining the type of anemia. Results: The most common types were IDA (61.9%) and IDWA (64.3%) in pAPS patients. In contrast, ACD was equally distributed across the three groups, with prevalences of 32%, 32%, and 36% (pAPS, sAPS, and SLE, respectively). A higher frequency of thrombosis was significantly associated with a high ferritin level ≥100 (*p* = 0.017) and high IL6 levels (*p* = 0.033) as well as fetal losses (*p* = 0.034 and *p* = 0.019, respectively). The logistic regression model identified ferritin as the only significant predictor of IDA (*p* = 0.023). For IDWA, both ferritin (*p* = 0.017) and hepcidin (*p* = 0.038) were significant predictors of this type of iron depletion. IL-6 levels were significantly correlated with ferritin and hsCRP levels (*p* = 0.004 and *p* = 0.007, respectively). In contrast, hepcidin did not show a statistically significant correlation with inflammatory markers. A total of 40% of patients with IDA had hepcidin levels below 10, and 48% of those with ACD had hepcidin levels above 10 (*p* = 0.036). Conclusions: It was found that iron deficiency anemia was the most common form in pAPS, while anemia of chronic disease was equally present across all patient groups. Ferritin emerged as an independent marker for identifying iron deficiency anemia in APS patients. Although hepcidin reflects a low-inflammatory state in APS, it proved to be a more valuable tool than ferritin in distinguishing the type of anemia, especially when ferritin levels were inconclusive. Clinical manifestations in APS patients correlated with inflammatory markers. Liver function or any drug used alone or in combination had no impact on anemia type.

## 1. Introduction

Antiphospholipid syndrome (APS) is a low-inflammatory thrombotic disease [1]. It can persist alone (primary APS—pAPS) or with another associated disease (secondary APS—sAPS), predominantly systemic lupus erythematosus (SLE) [2]. Anemia is often found in pAPS and may be the consequence of bleeding (10% of cases in catastrophic APS), hematinic deficiency (such as the deficiency of iron, B12, or folates), or hemolysis (within thrombotic microangiopathic syndromes or autoimmune hemolytic anemia) [3], with data on its actual prevalence lacking in the literature. Iron deficiency anemia (IDA) is more common in APS patients than in healthy individuals [4]. It can be the consequence of a significant reduction in vitamin C intake, which is attributed to the role of reactive oxygen species, as well as of iron malabsorption [4,5]. Also, anemia is found in about 50% of patients diagnosed with SLE. Its causes vary, and it can be the result of iron deficiency (since the patients are younger and of reproductive age) or autoimmune hemolysis (which is also one of the criteria for SLE diagnosis), and 60–80% of patients have anemia of chronic disease (ACD) [6]. Aspirin is a frequent treatment option, on its own or in combination with an anticoagulant, and may contribute to gastrointestinal tract (GIT) bleeding.

The two crucial regulatory molecules in systemic iron metabolism are the hepcidin-25 isoform and ferritin [7]. Hepcidin is only synthesized in the liver and is found in plasma. It binds to the enterocyte iron exporter, ferroportin, causing its internalization and degradation. Increased iron stores and inflammation induce hepcidin synthesis and thus lower the serum iron level. Ferritin is expressed in nearly all cells in the human body, serving as the primary iron storage protein, but also protects organs from damage caused by the potential toxicity of this metal. It is especially abundant in the liver (hepatocytes) and immune cells (macrophages), which are key sites for iron regulation and recycling. These cells, particularly macrophages and hepatocytes, also secrete ferritin into the bloodstream, making it a crucial indicator of iron levels. Ferritin expression is up-regulated by iron depletion and pro-inflammatory cytokines. Hyperferritinemia is more common among patients with APS than healthy individuals, especially among patients with CAPS [8]. Therefore, ferritin alone seems to not be a completely reliable indicator of iron stores in APS.

Key antiphospholipid antibodies in APS [2]—lupus anticoagulant (LA) and anticardiolipin antibodies (aCl)—are primarily produced by the immune system (B-cells and plasma cells). On the contrary, beta2glycoproteinI antibodies are predominantly synthesized in the liver, the same organ that has a crucial role in iron metabolism.

Assessing the etiology of anemia in patients with APS can be challenging. In this study, we compared the usefulness of iron metabolism markers (serum iron—Fe; total iron-binding capacity—TIBC; ferritin; hepcidin) in the presence of inflammatory markers, such as high-sensitivity CRP (hsCRP) and IL6, as a diagnostic method in determining the type of anemia in pAPS, sAPS, and SLE (excluding hemolytic anemia).

## 2. Materials and Methods

### 2.1. Study Population

This was a cross-sectional study involving 163 patients who were followed up as outpatients at the University Clinical Center Bezanijska kosa between June 2022 and June 2024. APS was determined based on the 2006 Sydney International Consensus Criteria [2], while SLE was established according to the Systemic Lupus International Collaborating Clinics Classification Criteria for Systemic Lupus Erythematosus (SLICC 2012) [9]. This study received approval from the local Ethics Committee (University Clinical Center Bezanijska kosa, as part of a PhD thesis; no number available) and fulfilled the ethical guidelines of the most recent Declaration of Helsinki (Edinburgh, 2000).

Exclusion criteria: patients with other inflammatory conditions, hemolytic anemia, acute or chronic liver disease, renal insufficiency, or malignancy; pregnant and breastfeeding patients; patients who had been on iron supplementation in the last 6 months; recent APS and/or SLE diagnosis (≤12 months); SLE flare and those older than 60 years.

### 2.2. Assessment of Iron Metabolism Parameters

Laboratory tests were collected in the morning (8–9 AM) during a regular follow-up visit. Patients were included consecutively if they fulfilled study cohort inclusion/exclusion criteria.

Peripheral venous blood samples were collected for iron profile (ferritin, iron, and TIBC), hsCRP, liver parameters (bilirubin total, bilirubin direct, aspartate (AST) and alanine aminotransferase (ALT), alkaline phosphatase (AP), and gamma GT (gGT), and de Ritis ratio (AST/ALT) was calculated), and renal parameters (urea, creatinine, and potassium), along with others (protein, albumin, serum amylases, and lactate dehydrogenase), using a Cobas Integra 400 Plus clinical analyzer (Roche Diagnostics). Complete blood count was tested immediately from the tube containing EDTA as an anticoagulant. Transferrin saturation (TSAT) was calculated as follows: TSAT % = iron [µg/dL]/TIBC [µg/dL] × 100.

The World Health Organization (WHO) defines the normal hemoglobin (Hgb) level cutoff as 130 g/L for males and 120 g/L for non-pregnant females [10]. Ferritin levels below 12–15 μg/L in adults are indicative of iron deficiency (ID) [6,11]. However, in clinical practice, iron deficiency can generally be diagnosed when ferritin levels fall below 30 μg/L [12]. TSAT levels under 15–20% are also indicative of ID. In chronic inflammatory conditions, when ferritin levels range from 100 μg/L to 300 μg/L, TSAT is recommended for diagnosing ID [13].

The cohort was categorized into four patient groups based on anemia parameters as follows:

- No anemia: normal hemoglobin (Hgb), ferritin ≥12 μg/L, and TSAT ≥15%.
- Iron deficiency without anemia (IDWA): normal Hgb, ferritin <12 μg/L, and normal or <15% TSAT.
- Iron deficiency anemia (IDA): low Hgb, ferritin <12 μg/L, and TSAT <15%.
- Anemia of chronic disease (ACD): low Hgb, ferritin >12 μg/L, and normal or >15% TSAT [14].

Serum samples for hepcidin measurement were stored at −70 °C until analysis and later assessed using the Sandwich ELISA method (DRG Diagnostics, NJ, USA). Reference ranges were as follows: 4.1–11.43 ng/mL for premenopausal women; 8.5–23.71 ng/mL for postmenopausal women; while the male median was 21.76 ng/mL [15]. When the hepcidin level was <10 ng/mL (3.6 pmol/L), the maximum percentage of correct classification was achieved for all ferritin cutoffs of iron deficiency [16].

Serum IL-6 was measured using the ELISA method. The pooled estimated reference range for IL-6 was 5.186 pg/mL (95% confidence interval [CI]: 4.631, 5.740) [17].

### 2.3. Assessment of Antiphospholipid Antibodies

As per consensus guideline recommendations, screening for LA was based on the use of two different screening tests: diluted activated partial thromboplastin time and sensitive activated partial thromboplastin time [18]. Anticardiolipin (aCL: IgG/IgM) and anti-β2glycoprotein I (β2GPI: IgG/IgM) antibodies were measured by an enzyme-linked immunosorbent assay (ELISA Binding Site) and expressed in G phospholipid (GPL) or M phospholipid (MPL) units (GPL-U and MPL U) in a single core laboratory. Cutoff levels for positivity were defined locally according to the manufacturer’s instruction as follows: B2-glycoprotein IgG antibody positivity—cutoff 12 PL-IgG-U/mL; B2-glycoprotein IgM antibody positivity—cutoff 12 PL-IgM-U/mL; anticardiolipin IgG antibody positivity—cutoff 20 PL-IgG-U/mL; anticardiolipin IgM antibody positivity—cutoff 20 PL-IgM-U/mL.

### 2.4. Statistical Analysis

Prevalence rates between groups were compared using the chi-square test and Fisher’s exact test (two-tailed), as appropriate. Continuous variables were expressed as means with standard deviations (SDs) and were compared between groups using Student’s *t*-test (two-tailed). Correlations between variables were assessed using Spearman’s rank correlation analysis. Multiple regression analysis was employed to determine the inter-relationships between two or more variables. A *p*-value of < 0.05 was considered statistically significant. The statistical SPSS 21.0 program was used for data analysis.

## 3. Results

The study cohort comprised 79 patients with pAPS, 47 with APS associated with SLE (sAPS) and 37 patients diagnosed with SLE. The mean age of APS patients was 50.6 ± 13.4 years, while the mean age of the SLE patients was 51.4 ± 16.9 years. There was no statistically significant difference between APS patients and SLE patients regarding age (*p* = 0.063) and gender (*p* = 0.057).

Patients with pAPS had a mean disease duration of 11.3 ± 5.1 years, and those with sAPS had a mean disease duration of 15.4 ± 8.1 years, while in patients with SLE, it was 11.9 ± 9.2 years. At the time of venipuncture, the mean SLEDAI score was 4.3 ± 2.7 (median 3.5). Thrombotic events occurred in 60.3% of pAPS patients and 74.5% of sAPS patients (*p* = 0.105). Obstetric events were observed in 51.3% of pAPS patients and 48.9% of sAPS patients (*p* = 0.799). B2-glycoprotein IgG antibody positivity (cutoff 12 PL-IgG-U/mL) was found in 6.3% of pAPS patients, 10.6% of sAPS patients, and 2.7% of SLE patients (*p* = 0.347). B2-glycoprotein IgM antibody positivity (cutoff 12 PL-IgM-U/mL) was observed in 12.7% of pAPS patients, 25.5% of sAPS patients, and 5.4% of SLE patients (*p* = 0.027). Anticardiolipin IgG antibody positivity (cutoff 20 PL-IgG-U/mL) was present in 3.8% of pAPS patients, 12.8% of sAPS patients, and 0% of SLE patients (*p* = 0.025). Anticardiolipin IgM antibody positivity (cutoff 20 PL-IgM-U/mL) was found in 30.4% of pAPS patients, 31.9% of sAPS patients, and 8.1% of SLE patients (*p* = 0.020). Lupus anticoagulant (LA) positivity was observed in 64% of pAPS patients, 57.4% of sAPS patients, and 2.3% of SLE patients (*p* = 0.001). There was no statistically significant difference regarding aPL presence between PAPS and sAPS patients (*p* > 0.05). None of the SLE patients had a history of thrombosis or obstetric events. Most of the patients were treated with drug combinations (77.9%). An oral anticoagulant (warfarin) was used by 96 out of 125 APS patients (76.8%), while acetylsalicylic acid (ASA) was used by 31.2% (39/125) and 45.9% (17/37) of SLE patients. Hydroxychloroquine (HQO) was used by 77.8% (98/126) of APS and 91.9% (34/37) of SLE patients. Prednisone was used by 42.6% (20/47) of sAPS patients and 43.2% (16/37) of SLE patients (Tables S1 and S2).

Patients with liver or renal impairments were not included in the study group. There were no statistically significant differences considering values of total bilirubin (*p* = 0.762), direct bilirubin (*p* = 0.313), AST (*p* = 0.103), ALT (*p* = 0.190), AP (*p* = 0.508), gGT (*p* = 0.053), and de Ritis ratio (*p* = 0.597) between pAPS/sAPS/SLE groups. Also, there were no statistically significant differences between liver parameters and any antiphospholipid antibody presence (*p* > 0.05). There were no statistically significant differences considering values of urea (*p* = 0.777) and creatinine (*p* = 0.170) in these groups.

### 3.1. Iron Metabolism Parameters and Type of Anemia

Based on the analyzed parameters, statistically significant differences were observed in TIBC (*p* = 0.029), hepcidin (*p* = 0.024), and IL-6 (*p* = 0.001) levels among the pAPS, sAPS, and SLE groups (Table 1).

Lower hemoglobin levels (IDA + ACD) were observed in 46 patients (28.2%) across the entire cohort (*p* = 0.001). However, there were no statistically significant differences in the presence of any type of anemia (IDA + IDWA + ACD) among the groups: 30 had anemia in pAPS (38%), 16 in sAPS (34%), and 14 in SLE (37.8%); (*p* = 0.897). Among the 60 patients with anemia (IDA + IDWA + ACD), the most common types were IDA + IDWA in those with pAPS (22/60; 36.7%) (Table 2). Among the 25 patients with ACD, there were 8 patients in the pAPS group (32%), 8 patients in the sAPS group (32%) and 9 patients in the SLE group (36%).

There were no statistically significant differences between the types of anemia and the presence of β2GPI IgG (*p* = 0.356), β2GPI IgM (*p* = 0.087), anticardiolipin IgG (*p* = 0.289), and anticardiolipin IgM (*p* = 0.323) antibodies or lupus anticoagulant (LA) positivity (*p* = 0.200). There were no statistically significant differences between the types of anemia and liver parameters: AST (*p* = 0.604), ALT (*p* = 0.984) or de Ritis ratio (*p* = 0. 767).

There were no statistically significant differences between the types of anemia and drug used—warfarin (*p* = 0.140), ASA (*p* = 0.187), HQO (*p* = 0.759) and prednisone (*p* = 0.862)—or any drug combination (*p* = 0.736).

Almost half of APS patients with thrombosis did not have anemia (59/125; 47.2%). However, thrombosis was also observed in 8.8% of patients with IDA, 1.6% with IDWA, and 8.0% with ACD and correlated to anemia presence (Spearman −0.229; *p* < 0.010). Patients without anemia had more thrombotic events (59/78% vs 23/28%; *p* = 0.011). Also, among the 125 APS patients, fetal loss occurred in 8.0% of patients with IDA, 8.0% with IDWA, and 8.8% with ACD (Table 3) and correlated with anemia presence (Spearman 0.277; *p* < 0.002). The statistical difference was significant (*p* = 0.002) between patients with anemia who had (31/68.9%) or did not have (14/31.1%) fetal loss.

**Figure 1:**
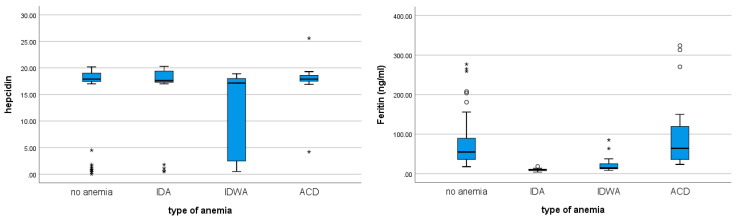
Hepcidin and ferritin levels depending on the type of anemia.

Although hepcidin and ferritin levels showed a statistically significant correlation (Spearman’s ρ = 0.159; *p* = 0.048), only ferritin values differed significantly among the groups (*p* < 0.001), while hepcidin did not (*p* = 0.065) (Table 4, Figure 1). Ferritin levels were also significantly correlated with the type of anemia (Spearman’s ρ = −0.272; *p* < 0.001).

Although patients with ACD had the highest hepcidin levels, the correlation between hepcidin levels and anemia type was not statistically significant (Spearman’s ρ = −0.105; *p* = 0.182). However, 40% of patients with IDA had hepcidin levels below 10, and 48% of those with ACD had hepcidin levels above 10 (*p* = 0.036). Additionally, among patients with ferritin levels <12, 60% had hepcidin levels <10 (*p* = 0.049).

The logistic regression model identified ferritin as the only significant predictor of IDA (B = −0.360, *p* = 0.023; 95% CI: 0.512–0.951). For IDWA, both ferritin (B = −0.066, *p* = 0.017; 95% CI: 0.886–0.988) and hepcidin (B = −0.167, *p* = 0.038; 95% CI: 0.722–0.991) were significant predictors of this type of iron depletion.

A ferritin level greater than 100 was significantly associated with a higher frequency of thrombosis (*p* = 0.017) and fetal loss (*p* = 0.034).

### 3.2. Markers of Inflammation

Elevated IL-6 levels were most commonly observed in patients with ACD (38.7%) (Table 5).

IL-6 and C-reactive protein (hsCRP) levels showed a statistically significant correlation (Spearman’s ρ = 0.410; *p* < 0.001). IL-6 levels were also significantly correlated with ferritin (Spearman’s ρ = 0.225; *p* = 0.004), as were hsCRP levels (Spearman’s ρ = 0.331; *p* = 0.007). In contrast, hepcidin did not show a statistically significant correlation with inflammatory markers.

The logistic regression model identified ferritin as the only significant predictor of elevated IL-6 levels (B = 0.023, *p* = 0.005; 95% CI: 1.007–1.040).

IL-6 levels were significantly higher in patients who experienced thrombotic events (*p* = 0.033) and in those with fetal losses (*p* = 0.019). Similarly, hsCRP levels were elevated in patients with thrombotic APS manifestations (*p* = 0.044) but not in those with fetal losses (*p* = 0.73).

## 4. Discussion

This cross-sectional observational study aimed to explore the relationships between hepcidin levels and a comprehensive set of variables related to anemia, iron metabolism, and inflammation in patients with APS and SLE, with the goal of better characterizing the type of anemia present. Among the 163 patients included, 60 (36.8%) were diagnosed with one of the following anemia types: IDA, IDWA, or ACD. There were no statistically significant differences in the overall prevalence of anemia (IDA + IDWA + ACD) among the groups, with up to 40% of patients in each group affected. However, IDA (61.9%) and IDWA (64.3%) were most frequently observed in patients with pAPS, while ACD was similarly distributed across all three groups (32%, 32%, and 36%, respectively).

Although hemoglobin cutoff values are useful for identifying anemia, their limitations should be acknowledged, and patients should be evaluated individually [7]. Our findings indicate that in both anemic and non-anemic APS patients, hepcidin correlates, albeit to varying degrees, with markers of anemia, iron metabolism, and inflammation. Moreover, hepcidin is a more effective discriminator of anemia type, as compared to ferritin. Additionally, clinical manifestations of APS were associated with the presence of anemia, suggesting that effective management of anemia may contribute to the prevention of these complications.

Anemia is a common and clinically significant comorbidity in patients with antiphospholipid syndrome (APS), arising from diverse etiological factors. However, data on its prevalence in this population remain limited. It is still unclear whether anemia in APS serves merely as a marker of low-grade inflammation or actively contributes to disease complications. This uncertainty has implications for treatment decisions.

Potentially treatable types of anemia include iron deficiency anemia (IDA) and iron deficiency without anemia (IDWA). The treatment approach depends on whether the iron deficiency is absolute—characterized by decreased total body iron—or functional, as in anemia of chronic disease (ACD), where iron stores are adequate or elevated but inaccessible to tissues due to sequestration.

A study by Klack et al. reported a higher incidence of IDA and IDWA in patients with primary APS (pAPS) compared to healthy controls, attributing this to factors such as hypermenorrhea and inadequate intake of folic acid and vitamin C [4]. Although an increased risk of gastrointestinal (GI) malignancy has not been observed in APS patients as of yet [19], treatment with acetylsalicylic acid (ASA) and oral anticoagulants may exacerbate subclinical GI bleeding, contributing to iron loss.

In systemic lupus erythematosus (SLE), anemia affects approximately 50% of patients and is multifactorial. The most frequent causes include ACD, IDA, autoimmune hemolytic anemia (AHA), anemia related to chronic renal insufficiency, and cyclophosphamide-induced myelotoxicity—often occurring in combination. Iron deficiency in SLE is common and is often due to menorrhagia, particularly in women of reproductive age, as well as increased GI blood loss associated with the use of non-steroidal anti-inflammatory drugs (NSAIDs), aspirin, and oral anticoagulants [6].

Ferritin and hepcidin are key regulators of iron metabolism and are commonly used in the evaluation of anemia types [7]. Iron is stored primarily in the form of ferritin, which can be efficiently mobilized when needed. In the absence of inflammation or chronic disease, serum ferritin levels correlate well with total body iron stores. However, ferritin is also an acute-phase reactant and can be nonspecifically elevated during inflammatory states, making it difficult to distinguish between absolute and functional iron deficiency (ID). In such cases, the diagnostic cutoff for ferritin levels is often raised to 100 μg/L to account for chronic inflammation.

Moderate hyperferritinemia has been observed in various autoimmune diseases, including systemic lupus erythematosus (SLE), rheumatoid arthritis (RA), multiple sclerosis (MS), and antiphospholipid syndrome (APS) [20]. Inflammatory processes—particularly those involving the IL-6 signaling pathway—stimulate hepcidin production, leading to increased iron sequestration, reduced intestinal iron absorption, and inhibition of iron release from macrophages. As a result, both serum iron and transferrin levels decrease, and total iron-binding capacity (TIBC) is reduced—features characteristic of anemia of chronic disease (ACD) [21].

Importantly, hepcidin measurement can help differentiate ACD from iron deficiency anemia (IDA) in patients with overlapping features. This diagnostic distinction has been demonstrated in conditions such as tumor-associated anemia [22], rheumatoid arthritis [23], inflammatory bowel disease [24], and critical illness [25].

In this study, there was no statistically significant difference in ferritin levels among the pAPS, sAPS, and SLE groups, suggesting a uniformly low state of inflammation across all patient groups. Also, hepcidin and ferritin levels were positively correlated. However, hepcidin levels differed significantly among the groups (*p* = 0.024), with the pAPS group showing significantly lower values compared to both the sAPS and SLE groups (*p* < 0.001). This was not expected due the fact that inflammation in SLE drives hepcidin production and could be explained by the influence of anemia on hepcidin level.

When analyzing only the anemic patients, ferritin levels differed significantly among anemia subgroups (*p* < 0.001), whereas the difference in hepcidin levels did not reach statistical significance (*p* = 0.065). Ferritin levels also showed a significant correlation with anemia type (Spearman’s ρ = −0.272; *p* < 0.001), with the lowest values observed in patients with IDA. These findings are expected and do not substantially advance current understanding of anemia, which indicates the need to include other markers of anemia to determine its type. Although patients with ACD had the highest hepcidin levels, the correlation between hepcidin and anemia type was not statistically significant (Spearman’s ρ = −0.105; *p* = 0.182). However, 40% of patients with IDA had hepcidin levels below 10 ng/mL, while 48% of those with ACD had hepcidin levels above 10 ng/mL (*p* = 0.036). Additionally, among the patients with ferritin levels below 12 ng/mL, 60% had hepcidin levels below 10 ng/mL (*p* = 0.049).

Logistic regression analysis confirmed that ferritin was the only significant predictor of IDA (B = −0.360, *p* = 0.023; 95% CI: 0.512–0.951). For IDWA, both ferritin (B = −0.066, *p* = 0.017; 95% CI: 0.886–0.988) and hepcidin (B = −0.167, *p* = 0.038; 95% CI: 0.722–0.991) were identified as significant predictors of iron depletion.

C-reactive protein (CRP) levels are typically not significantly elevated in primary APS alone [26]. However, mild elevations can occur in the presence of underlying inflammation, infection, or coexisting autoimmune conditions. While CRP can contribute to endothelial dysfunction, it is not considered a reliable predictor of thrombosis in APS [27]. In contrast, CRP levels tend to be more markedly elevated in secondary APS (sAPS), particularly in patients with SLE. In these cases, CRP levels often correlate with both disease activity and cardiovascular risk factors, reflecting a two-way relationship between inflammation and vascular risk [28].

Interleukin-6 (IL-6) levels are typically moderately elevated in primary APS (pAPS) but can be significantly higher in sAPS, particularly during disease flare-ups or periods of active inflammation. IL-6 plays an important role in promoting endothelial activation, tissue factor expression, and platelet activation, thereby contributing to vascular injury and increased thrombotic risk. In systemic lupus erythematosus (SLE), IL-6 is a key mediator of disease pathogenesis and has also been shown to correlate with anemia in affected patients [29].

Consistent with the existing literature, this study found no statistically significant differences in hsCRP levels among the pAPS, sAPS, and SLE groups. The presence of statistically significant differences in IL-6 without corresponding differences in hsCRP, given that hsCRP reflects IL-6 activity indirectly, could be explained by the so-called “SLE paradox”—IL-6 receptor shedding—which is in accordance with a recent study [30]. However, hepcidin levels were significantly higher in patients with sAPS and SLE. Regarding anemia subtypes, elevated IL-6 levels were most commonly observed in patients with anemia of chronic disease (ACD) (38.7%), as were elevated hsCRP levels (33.3%). Ferritin, acting as an acute-phase reactant, followed a similar pattern and correlated with inflammatory activity, unlike hepcidin, which did not show a consistent association with inflammation.

According to the existing literature, iron deficiency anemia is a well-established risk factor for thrombotic events and maternal mortality in the general population. However, data on this association in patients with antiphospholipid syndrome remain limited [31,32]. Additionally, anemia of chronic disease is frequently observed in inflammatory diseases and is often present alongside thrombotic events [33]. Elevated IL-6 levels, an upstream driver of the complement system, may contribute to this association. This could help explain the significantly higher IL-6 levels observed in patients with a history of fetal loss.

In our study, a statistically significant difference was found between APS clinical manifestations and the presence of anemia. Thrombotic events occurred in 28% of patients with anemia, with similar prevalence among those with IDA (13.4%) and ACD (12.2%). Fetal loss was observed in 49.2% of anemic patients, regardless of the anemia type.

Consistent with previous reports, other evaluated parameters also provided insight into the risk of APS-related clinical manifestations [34,35]. Thrombotic events, as clinical manifestations of APS, were significantly more frequent in patients with ferritin levels >100 ng/mL. Additionally, IL-6 levels were significantly higher in patients who experienced thrombotic events (*p* = 0.033) than in those with fetal losses (*p* = 0.019). Elevated hsCRP levels were also associated with thrombotic manifestations (*p* = 0.044) but not with fetal losses (*p* = 0.73). Since IL6 is a key pro-inflammatory cytokine, it promotes hepcidin, CRP and ferritin up-regulation. It shows varied correlations across studies, suggesting complex interactions with other inflammatory and clotting factors as contributing factors in the development of the APS clinical presentation [34].

The liver is the only place where beta2glycoproteinI synthesis takes place and also the regulatory organ of iron metabolism. According to literature data, levels of β2GPI protein negatively correlate with liver function tests (bilirubin and AST/ALT ratio), suggesting that β2GPI protein levels are reduced in patients with chronic liver diseases and progressively decline with increasing disease severity [36,37]. APS is associated with a greater proportion of β2GPI being in an oxidized state [38]. β2GPI becomes antigenic upon oxidization in the absence of the antioxidant protein, PON1 (paraoxonase 1) [39]. Rapamycin treatment in vivo restores PON1 secretion and protects blood antigens such as β2GPI from oxidation [39]. In this study, rapamycin (mTOR inhibitor) and antioxidants were not used as treatment options. We analyzed liver biochemistry panels and found that liver function had no impact on β2GPI production within the normal liver function in these patients. Also, we did not notice any statistically significant differences between the types of anemia and liver parameters. As mentioned above, treatment could have an impact on the presence of anemia in APS and SLE patients. Most patients in the study (77.9%) were treated with a combination of drugs. There was no significant association between the presence of anemia and the four main drugs used (warfarin, ASA, HQO, and prednisone) or any drug combination.

Limitation of the study: Many of the observed results could occur in patients without SLE or APS. Inclusion of a control group consisting of individuals with iron deficiency anemia and anemia of chronic disease without autoimmune disorders would strengthen the study.

## 5. Conclusions

This study is among the few that explore the prevalence of anemia in patients with APS and/or SLE. Also, we assessed the diagnostic value of iron metabolism markers—including Fe, TIBC, ferritin, and hepcidin—and their interplay with markers of inflammation. It was found that iron deficiency anemia was the most common form in pAPS, while anemia of chronic disease was equally present across all patient groups. Several results—such as ferritin indicating iron deficiency and hepcidin reflecting anemia of chronic disease—were expected based on established pathophysiology. However, although hepcidin reflects a low-inflammatory state in APS, it proved to be a valuable tool in distinguishing the type of anemia, especially when ferritin levels were inconclusive. Given that APS clinical manifestations were more frequent among patients with anemia, the results support the importance of recognizing and treating IDA/IDWA in pAPS. Additionally, effective management of systemic lupus erythematosus is essential to prevent ACD in patients with sAPS. Since the liver is the main site of beta2gpI and hepcidin synthesis and is a regulator of iron metabolism, it was shown that normal liver function in these patients had no effect on the production of beta2gpI antibodies or on hepcidin synthesis. No drug or treatment combination had a significant impact on the type of anemia found in APS/SLE patients.

## Figures and Tables

**Table 1:** Iron metabolism and inflammatory parameters.

Mean ± SD	pAPS	sAPS	SLE	*p*
Hgb (g/L)	131.6 ± 17.6	128.9 ± 11.3	126.9 ± 9.7	0.285
MCV (fl)	87.7 ± 4.6	88.4 ± 4.2	88.1 ± 5.1	0.860
Fe (µg/dL)	16.1 ± 8	15.1 ± 6.1	15.3 ± 6.5	0.727
TIBC (µg/dL)	74.6 ± 16.4	69.4 ± 12.2	67.5 ± 9.7	0.029
TSAT (%)	21.9 ± 9.1	22.6 ± 9.9	23.5 ± 11.2	0.853
Ferritin (ng/mL)	46.3	43.6	44.0	0.580
Hepcidin (ng/mL)	16.6 ± 4.9	15.6 ± 6.02	14.4 ± 6.9	0.024
hsCRP (mg/L)	1.65	1.40	1.65	0.449
IL6 (pg/mL)	1.21	1.95	1.89	0.001

Ferritin, hsCRP, IL6—median (Table S3: Biochemistry parameters).

**Table 2:** Patient groups based on anemia parameters.

*n* (%)	No Anemia	IDA	IDWA	ACD
pAPS	49 (62.0)	13 (16.5)	9 (11.4)	8 (10.1)
sAPS	31 (66.0)	5 (10.6)	3 (6.4)	8 (17.0)
SLE	23 (62.2)	3 (8.1)	2 (5.4)	9 (24.3)

**Table 3:** Clinical APS manifestations and anemia.

*n* (%)	No Anemia	IDA	IDWA	ACD	*p*
Thrombosis (*n* = 82)	59 (72.0)	11 (13.4)	2 (2.4)	10 (12.2)	0.002
Fetal loss (*n* = 63)	32 (50.8)	10 (15.9)	10 (15.9)	11 (17.5)	0.010

**Table 4:** Hepcidin and ferritin levels among groups.

	No Anemia	IDA	IDW	ACD	*p*
Hepcidin mean ± SD	16.6 ± 5.1	15.1 ± 7.1	12.0 ± 8.0	17.8 ± 3.3	0.065
Ferritin Med [min–max]	55.0 [63.1–84.3]	9.8 [8.5–11.6]	14.3 [11.9–38.1]	64.1 [59.1–130.4]	<0.001

**Table 5:** Markers of inflammation.

		No Anemia *n* (%)	IDA *n* (%)	IDWA *n* (%)	ACD *n* (%)	*p*
IL6 (pg/mL)	<5	88 (66.7)	19 (14.4)	12 (9.1)	13 (9.8)	0.001
	>5	15 (48.4)	2 (6.5)	2 (6.5)	12 (38.7)	
hsCRP (mg/L)	<5	85 (62.5)	21 (15.4)	14 (10.3)	16 (11.8)	0.003
	>5	18 (66.7)	0	0	9 (33.3)	

## Data Availability

The data presented in this study are available on request from the corresponding author due to ethical reasons.

## Supplementary Materials

The following supporting information can be downloaded at: https://www.mdpi.com/article/10.3390/diseases14030101/s1, Table S1: The prevalence of the most commonly drug used; Table S2: The prevalence of all drug combinations; Table S3: Biochemistry parameters.

## Abbreviations

The following abbreviations are used in this manuscript:

APS	Antiphospholipid syndrome
SLE	Systemic lupus erythematosus
IDA	Iron deficiency anemia
IDWA	Iron deficiency without anemia
ACD	Anemia of chronic disease
