# Supplementary material for: The Prevalence of Anemia and Diagnostic Usefulness of Ferritin and Hepcidin in Antiphospholipid Syndrome and Systemic Lupus Erythematosus Patients
Source: Diseases. 2026 Mar 11;14(3):101. doi: 10.3390/diseases14030101 (PMC13025763; doi:10.3390/diseases14030101)
Supplement: Supplementary file 1 [file diseases-14-00101-s001.zip › diseases-4170088-supplementary.pdf]

Table S1: The prevalence of the most commonly drug used

N/%	PAPS	SAPS	SLE	Total
**warfarin**	55/57.3	41/42.7	0/0	96/100
**ASA**	32/57.1	7/12.5	17/30.4	56/100
**HQO**	57/43.2	41/31.1	34/25.8	132/100
**Prednisone**	0/0	20/55.6	16/44.4	36/100
**Total N**	79	47	37	163

Table S2: The prevalence of all drug combinations

		PAPS	SAPS	SEL	Total
**Without treatment**	N	0	0	2	2
	% within all	0.0%	0.0%	100.0%	100.0%
	% within dg	0.0%	0.0%	5.4%	1.2%
**warfarin**	N	17	1	0	18
	% within all	94.4%	5.6%	0.0%	100.0%
	% within dg	21.5%	2.1%	0.0%	11.0%
**ASA**	N	2	1	0	3
	% within all	66.7%	33.3%	0.0%	100.0%
	% within dg	2.5%	2.1%	0.0%	1.8%
**HQO**	N	1	0	11	12
	% within all	8.3%	0.0%	91.7%	100.0%
	% within dg	1.3%	0.0%	29.7%	7.4%
**pronisone**	N	0	0	1	1
	% within all	0.0%	0.0%	100.0%	100.0%
	% within dg	0.0%	0.0%	2.7%	0.6%
**warfarin+ASA**	N	3	0	0	3
	% within all	100.0%	0.0%	0.0%	100.0%
	% within dg	3.8%	0.0%	0.0%	1.8%
**warfarin+HQO**	N	29	22	0	51
	% within all	56.9%	43.1%	0.0%	100.0%
	% within dg	36.7%	46.8%	0.0%	31.3%
**ASA+HQO**	N	21	2	9	32
	% within all	65.6%	6.3%	28.1%	100.0%
	% within dg	26.6%	4.3%	24.3%	19.6%
**ASA+pronisone**	N	0	1	0	1
	% within all	0.0%	100.0%	0.0%	100.0%
	% within dg	0.0%	2.1%	0.0%	0.6%
**warfarin+ASA+HQO**	N	6	1	0	7
	% within all	85.7%	14.3%	0.0%	100.0%

	% within dg	7.6%	2.1%	0.0%	4.3%
**ASA+HQO+ pronisone**	N	0	2	9	11
	% within all	0.0%	18.2%	81.8%	100.0%
	% within dg	0.0%	4.3%	24.3%	6.7%
**warfarin+pronisone**	N	0	3	0	3
	% within all	0.0%	100.0%	0.0%	100.0%
	% within dg	0.0%	6.4%	0.0%	1.8%
**warfarin+HQO+ pronisone**	N	0	14	0	14
	% within all	0.0%	100.0%	0.0%	100.0%
	% within dg	0.0%	29.8%	0.0%	8.6%
**HQO+pronisone**	N	0	0	5	5
	% within all	0.0%	0.0%	100.0%	100.0%
	% within dg	0.0%	0.0%	13.5%	3.1%
**Total**	N	79	47	37	163
	% within all	48.5%	28.8%	22.7%	100.0%
	% within dg	100.0%	100.0%	100.0%	100.0%

N - count
ASA, acetylsalicylic acid; HQO, hydroxychloroquine;

Table S3: Biochemistry parameters

Parameter<br>Normal range, units	PAPS/72 pts	SAPS/45 pts	SLE/36 pts	p
Urea<br>(2.8 – 8.3 mmol/L)	4.4±1.5<br>1.6-8.3	4.7±1.4<br>2.5-10.4	4.7±1.4<br>2.1-8.7	0.777
Creatinine<br>(49–110 µmol/L)	69.5±14.9<br>36-120	75.3±17.9<br>35-127	68.0±11.6<br>46-109	0.150
Potassium<br>(3.5-5.1 mmol/L)	4.4±0.4<br>3.4-5.2	4.3±0.4<br>3.4-5.4	4.3±0.5<br>2.1-5.2	0.667
Bilirubin total<br>(<21 µmol/L)	8.1±4.4<br>1.5-32.2	9.8±8.0<br>2.7-52.4	8.9±5.6<br>3.1-35	0.762
Bilirubin direct<br>(<21 µmol/L)	2.1±0.9<br>0.7-6.5	2.5±1.8<br>1.1-12	2.6±1.4<br>1-6.3	0.313
AST<br>(<34 U/L)	19.7±7.9<br>12-73	18.6±5.3<br>11-38	19±9.3<br>11-62	0.103
ALT<br>(10-49 U/L)	19.2±9.2<br>9-67	18±8.4<br>8-43	18.9±10.9<br>8-61	0.190
De Ritis Ratio	1.1±0.2<br>0.46-1.78	1.1±0.3<br>0.47-1.86	1.1±0.5<br>0.21-3.14	0.597
Alkaline phosphatase<br>(46-116 U/L)	57.8±16.6<br>26-94	55.9±15.9<br>27-91	59.3±17.3<br>30-96	0.508
Gama GT	19.9±8.2	20.4±8.2	16.9±5.0	0.053

(6-42 U/L)	5-37	6-40	10-31	
Protein<br>(57-82 gr/L)	71.9±4.5<br>61-82	70.2±4.5<br>59-80	69.5±9.7<br>35-81	0.232
Albumin<br>(35-52 gr/L)	43.7±3.5<br>32-51	43.7±3.3<br>36-50	44.1±2.7<br>39-50	0.822
Uric acid<br>(184-464 μmol/L)	247.2±68.4<br>78-463	267.8±71.3<br>92-417	251.3±65.3<br>134-461	0.237
Serum amylase<br>(30-118 U/L)	63±19.7<br>25-109	68.8±22.6<br>17-119	49.4±18.3<br>16-102	0.129
LDH<br>(257-526 U/L)	308.8±55.4<br>148-417	305.7±58.9<br>169-445	307.3±60.4<br>218-459	0.734
Glucosa<br>(4.1-5.9 mmol/L)	5.0±0.7<br>3.5-8.2	4.9±0.7<br>3.8-7.1	4.9±0.6<br>4.2-7.2	0.258

LDH= lactate dehydrogenase, De Ritis Ratio=AST/ALT
